# Towards meaningful inclusion of people affected by TB: lessons from the COVID-19 response

**DOI:** 10.5588/ijtld.22.0058

**Published:** 2022-06-01

**Authors:** A. Versfeld, J. Malar, V. Soltan, B. Kampoer, R. Mburu, C. Nawina, O. Khuat

**Affiliations:** 1 Department of Anthropology, University of Cape Town, Cape Town, South Africa; 2 Stop TB Partnership, Geneva, Switzerland; 3 Dynamique de la Réponse d’Afrique Francophone sur la Tuberculose (DRAF TB), Yaounde, Cameroon; 4 WACI Health, Nairobi, Kenya; 5 Community Initiative for Tuberculosis, HIV/AIDS and Malaria plus related diseases (CITAMPlus), Lusaka, Zambia; 6 Center for Support Community Development Initiatives (SCDI), Hanoi, Vietnam

In 2018, the United Nations High-Level Meeting (UNHLM) on TB countries committed to “transform the TB response to be equitable, rights-based and people-centred”.^1^ Yet people affected by TB remain largely marginalised in the design, implementation and assessment process for the TB response.^2^ This marginalisation is harmful; people affected by TB are experts about their own lives and needs and their meaningful inclusion is not just a right, it is an imperative for a more effective TB response. Changing the status quo is more important now than ever. The COVID-19 pandemic has had a devastating impact on the TB response: in 2020, 18% fewer people were diagnosed with TB than in 2019, and best estimates suggest that there were over 100,000 TB-related deaths.^3^ Progress has been set back an estimated 12 years and the world is not on track to achieve the 2018 UNHLM targets. Here, we present our work to develop, test and assess a new model for better inclusion of TB-affected people in TB governance. We suggest that this model has much to offer a more inclusive, efficient and rights-based TB response.

In 2020, The Global Fund (TGF) launched the COVID-19 Response Mechanism (C19RM) to provide support for eligible TGF-funded countries to respond to the COVID-19 pandemic; mitigate the impact of COVID-19 on programmes to fight HIV, TB, malaria; and strengthen systems for health.^4^ Funding guidelines stipulate that country co-ordinating mechanisms (CCMs) – the collectives responsible for the governance of TGF country processes – must engage with affected community and civil society and ensure that their perspectives and needs are incorporated throughout the grant cycle. Concerned by the historical marginalisation of people affected by TB, and noting a lack of precedent for modes of effective inclusion, the Stop TB Partnership (STP) collaborated with civil society groups in 18 high TB burden countries for the design and implementation of what became the “STP TB community C19RM support package”. In many countries, this was the first time there had been direct support for TB communities to mobilise, strategize, engage and advocate in TGF request processes. With support from USAID, the package included small grants of up to USD15,000 per organisation; guidance documents on TB and COVID-19 bi-directional interventions and on TB Communities, Rights and Gender (CRG) inclusion; virtual training sessions; in-country sensitisation meetings; technical assistance to writing teams; ongoing engagement and review of the C19RM grant requests by the STP Country and Community Support for Impact team; and online facilitation of South-to-South knowledge and capacity exchange. It was rapidly deployed and allowed flexibility in the terms of use.^5^

Despite challenges, including time limitations, digital exclusion of marginalised populations, COVID-19 restrictions and – in some cases – difficulties in accessing CCM deliberations, the impact of the support package exceeded our expectations. People affected by TB and other key and vulnerable populations were directly included funding request decision-making processes, often for the very first time. TB communities developed their knowledge and capacity related to TGF processes. High-level advocacy processes related to TB community priorities and CRG were set in motion. Ultimately, the support package led to C19RM funding requests featuring the concerns and priorities of the TB community, with some countries securing significant requests for investments in TB CRG activities (such as community system strengthening, stigma reduction, activation of human right and community-led monitoring). Finally, community-based strategies, priorities and agendas were set in the included countries, with potential long-lasting impact. None of this was to the detriment of a biomedical focus – all 18 country teams stood by the call to funders to leverage investments in COVID-19 to help end TB^1^ by emphasising the need for bi-directional TB and COVID-19 screening, treatment, diagnosis and care.

The meaningful inclusion of TB communities in grant-making requests is a long-term strategy for success. Inclusion means that community-related requests are constructed from first-hand accounts, gaining direction and accuracy. This also sets up long-term positive partnership opportunities and builds the willingness of affected communities to fully support grant activities. However, TB community partners in some countries struggled to access or influence CCMs. In other countries, despite support, TB-affected community groups lacked knowledge and experience to make structured inputs that were easily translated into country funding requests. In contrast, those countries where community partners were most successful had a longer history of support, for example, through Challenge Facility For Civil Society.^6^ Also, sometimes there was a low availability (or willingness) of organisations or government partners working on other pathologies such as COVID-19, HIV and malaria to collaborate with TB-affected communities. Nevertheless, through the TB CRG C19RM Support Package, the level of TB community engagement and influence in a TGF process was unprecedented. Our experience has demonstrated that supporting meaningful inclusion of TB communities is feasible, even with limited investment. In fact, the commitment and drive of TB communities means that dedicated, and direct investment in TB community system strengthening can reap benefits that are disproportionate to that investment. We cannot afford to not scale up investment that allows TB communities to better organise themselves, in ways that are inclusive of networks of TB survivors, to find solutions to matters that impact their lives. Inclusion of people affected by TB is a matter for action, not rhetoric, which needs to be evident in every aspect of the TB response.

Our work has highlighted some key elements of effective support for greater participation of TB communities. Investments and support should be ongoing processes of community systems strengthening, rather than one-off events, or financial injections. Support packages should be rapidly deployed, flexible, responsive to community needs, and coupled with technical assistance and South-South knowledge exchange. Ongoing technical support should be provided for planning, implementation, monitoring, evaluation, and governance of activities. These elements should become the standard for all TB programming, both domestic and donor funded. In October 2021, the STP Board committed to further efforts towards inclusion of people affected by TB. We call on others to do the same.
